# Impact of Health Research Systems on Under-5 Mortality Rate: A Trend Analysis

**DOI:** 10.15171/ijhpm.2016.147

**Published:** 2016-11-26

**Authors:** Bahareh Yazdizadeh, Mahboubeh Parsaeian, Reza Majdzadeh, Sima Nikooee

**Affiliations:** ^1^Knowledge Utilization Research Center, Tehran University of Medical Sciences, Tehran, Iran.; ^2^Epidemiology and Biostatistics Department, School of Public Health, Tehran University of Medical Sciences, Tehran, Iran.

**Keywords:** Under-Five Mortality Rate (U5MR), Research Impact Assessment, Research Payback, Research Contribution

## Abstract

**Background:** Between 1990 and 2015, under-5 mortality rate (U5MR) declined by 53%, from an estimated rate of 91 deaths per 1000 live births to 43, globally. The aim of this study was to determine the share of health research systems in this decrease alongside other influential factors.

**Methods:** We used random effect regression models including the ‘random intercept’ and ‘random intercept and random slope’ models to analyze the panel data from 1990 to 2010. We selected the countries with U5MRs falling between the first and third quartiles in 1990. We used both the total articles (TA) and the number of child-specific articles (CSA) as a proxy of the health research system. In order to account for the impact of other factors, measles vaccination coverage (MVC) (as a proxy of health system performance), gross domestic product (GDP), human development index (HDI), and corruption perception index (CPI) (as proxies of development), were embedded in the model.

**Results:** Among all the models, ‘the random intercept and random slope models’ had lower residuals. The same variables of CSA, HDI, and time were significant and the coefficient of CSA was estimated at -0.17; meaning, with the addition of every 100 CSA, the rate of U5MR decreased by 17 per 1000 live births.

**Conclusion:** Although the number of CSA has contributed to the reduction of U5MR, the amount of its contribution is negligible compared to the countries’ development. We recommend entering different types of researches into the model separately in future research and including the variable of ‘exchange between knowledge generator and user.’

## Background

One of the Millennium Development Goals (MDGs) was to ‘reduce child mortality’ and one of its targets was to “reduce by two-thirds, between 1990 and 2015, the under-five mortality rate.” Based on the World Health Organization (WHO) report, between 1990 and 2015, under-5 mortality rate (U5MR) declined by 53% from an estimated rate of 91to 43 deaths per 1000 live births, globally.^1^

However, given the importance of health research in the promotion of well-being in the world, this question comes to mind, “what is the share of countries’ health research systems in the reduction of U5MR?” To achieve health, research is essential for the advancement of technology, systems, and service delivery. In recent years, much emphasis has been laid on the role of research in health promotion. Research plays a significant role in empowering health systems for improving system performance and public health impact.^2^ The 2013 WHO report titled *“Research for Universal Health Coverage”* emphasizes the significance of achieving health through conducting research. The report states that through the utilization of research we must show which service deliveries we need; how to increase their coverage; how to improve financial risk protection; how to measure the aforementioned; and eventually, how to ensure that we have attained universal health coverage.^3^

Hence, measuring the impact of health research is very important. The impact of health research can be measured by two approaches, case studies and ecological studies.^4^ In case studies, a study or a group of studies is selected and their effects are examined over time. There are a limited number of models and indicators used to examine the effect of research through case studies, the most important of which is the ‘payback model,’ which measures health research impact in five domains including ‘knowledge advancement,’ ‘capacity building,’ ‘impact on decision-making,’ ‘health impact,’ and ‘social and economic impact.’^5,6^ In ecological studies, the variables used in the studies are collected and interpreted at a global level. For example, the relationship between the amount of expenditure on health research and the reduction of mortality from certain diseases is evaluated. One of the most important challenges in such studies is to find out the extent of attribution of health improvement to health research (attribution problem) in the presence of other interventions that may fall outside the field of health such as interventions targeted at education or well-being, or other variables such as political will, public opinion and industrial pressure.^7^

This study aimed to investigate the impact of the health research system on the U5MR trend with respect to other interventions and changes through an ecological approach.

## Methods

In order to achieve the goals of this study the following steps were taken:

### 1. Development of a Basic Model

Health improvement in countries can be attributed to the interventions inside and outside the health system. Health system performance and the level of a country’s development are critical in its health improvement. In order to reduce the ‘attribution problem,’ in addition to the status of health research systems in various countries, we considered a model in which other variables affecting health status were also present:

*Health Status ~ Health research system & health system performance & development of the country*

This model is based on the ‘demand for health capital’ (Grossman) model. Here, the presumption is that at individual level, health is dependent on inputs (nutrient intake, housing, income, recreation, consumption of public goods, education) and medical care. At macro level, the determinant factors of health are economic, social, and environmental factors.^8^ The model applied in this study is an analysis at macro level, wherein the level of development of countries is considered as a composite proxy of economic, social, and environmental factors. The ‘health system performance’ variable is considered an important factor in trends of health change, which has been implemented separately. Thus, in this study, in addition to the variables of the Grossman model, we have added the ‘research’ variable to predict the health status. Each component of this equation was given a proxy index or an appropriate combined indicator.

### 2. Determining Appropriate Indices

In this study, the total articles (TA) published in international databases and child-specific articles (CSA) were included as the performance indicators of the health research system. We did not exclude the editorial papers, letters, and commentaries, based on previous research, these type of papers contain more explicit messages, because they are written by expert researchers and offer a body of knowledge.^9^

Normally, various indicators evaluate health system performance. However, since one of the most important causes of reduction in the U5MR was the increased measles vaccination coverage (MVC), this index was included in our model as the proxy of health system performance. As mentioned in The MDGs report,^10^ between 2000 and 2008, the first and second doses of measles vaccination accounted for a 78% reduction in mortality caused by this disease in the world.

In order to account for the development of a country, a variety of indicators can be used, each with certain advantages and disadvantages. Two main criteria have been introduced for this purpose: gross domestic product (GDP) per capita and human development index (HDI). As presented in World Bank’s website: *“GDP per capita is gross domestic product divided by midyear population. GDP is the sum of gross value added by all resident producers in the economy plus any product taxes and minus any subsidies not included in the value of the product.”*^11^

According to the United Nations Development Program (UNDP), Human Development Reports*,* the* “HDI is a summary measure of average achievement in key dimensions of human development: a long and healthy life, being knowledgeable and having a decent standard of living.”* The health dimension is measured by life expectancy at birth; the education dimension is measured by mean years of schooling for adults aged 25 years and expected years of schooling for children of school-entering age, and the standard of living dimension is measured by gross national income per capita.^12^ We used both GDP and HDI indices in this study. Given the importance of corruption perception index (CPI) in a country’s administrative performance, this index was also included as a proxy of country development; Hunger, child mortality, illiteracy, and poverty cannot be eliminated inasmuch as corruption usurps the world’s most deprived countries’ resources.^13^

### 3. Country Selection

Countries were ranked by their U5MR from 1990 in ascending order, based on the data provided by the World Bank, and the aggregated frequency was calculated. Countries in the first to third quartiles were selected. These were selected because countries which are in a good health condition (higher than the third quartile) have not experienced significant variations in the U5MR over time. However, for the countries with poor conditions (lower than the first quartile), the reliability of data is questionable.

### 4. Data Collection

We searched PubMed to determine the number of articles published by each country. The search strategy was planned in a way that it could both specify the total number of health-related articles published by a country and the articles that were written with a particular focus on children. The time range was set to 1980 to 2010. To determine the TA published by each country, we searched the name of each country among the titles, MeSH terms, and authors’ affiliations (both in lowercase and uppercase letters) in ‘title and author’s affiliation.’ Meanwhile, due to the similarity of the names of some of the countries with other words the ‘NOT’ command was used to avoid those words in the search.

Using the keywords ‘child’ and ‘disease,’ and by adding ‘malnutrition’ to the list of diseases, ie, HIV/AIDS, diarrhea, pneumonia, malaria, measles, and neonatal disorders (with the exclusion of injuries/accidents), the number of CSA was calculated for each country. The search strategy is shown in online Supplementary file 1.

To obtain the required information about the dependent and independent variables, well-known databases were used.

The MVC (% of children aged between 12-23 months which have received measles vaccination), GDP per capita (constant US$2000), and U5MR from 1990 to 2010 were obtained from the World Bank website (http://data.worldbank.org).

The CPI was obtained from the ‘Transparency International’ website (http://www.transparency.org/), and HDI was extracted from the UNDP website (http://www.undp.org).

### Data Analysis

For each country, U5MR data for the five junctures of 1990, 1995, 2000, 2005, and 2010 were defined as dependent variables. For each point, the GDP, the MVC, the mean number of articles during a 5-year span before the aforementioned years, and the overall CPI mean over a period of 15 years were considered as independent variables (because of the missing data). The HDI for each year was attributed to the same year’s U5MR (for example, the HDI in 1990 was ascribed to the U5MR in 1990). MVC data were available only up to 2009. As a result, the mean of this index during the period between 2006 and 2009 was also generalized for 2010. The variables calculated for each juncture are shown in Table 1.

**Table 1:** Time-Adjustments of Variables Used in the Model

	**U5MR**	**TA/CSA/GDP**	**MVC**	**HDI**	**CPI**
**Time**	1990	Average of 1986-1990	Average of 1986-1990	1990	Average of 1996-2010
	1995	Average of 1991-1995	Average of 1991-1995	1995	
	2000	Average of 1996-2000	Average of 1996-2000	2000	
	2005	Average of 2001-2005	Average of 2001-2005	2005	
	2010	Average of 2006-2010	Average of 2006-2009	2010	

Abbreviations: TA: total articles; HDI: human development index; U5MR, under-five mortality rate; GDP, gross domestic product; MVC, measles vaccination coverage; CPI, corruption perception index; CSA, child-specific articles.

At first, we performed univariate analysis between the dependent variable (U5MR) and the independent variables. The variables that were significant at the *P* ≤ .2 level were included in the model.

Since the observations for each country are interdependent, random effects regression models were used to analyze the two models (‘random intercept model’ and ‘random intercept and random slope model’). In the random intercept model, the intercept can vary between countries. In the random intercept and random slope model, however, we extend the flexibility of the model to capture different slopes for each country for some important variables such as time. To improve the efficiency of the models, a centered variable was used for the variables of ‘total articles,’ ‘child-specific articles,’ and ‘time’ (the year 1990 was considered as the time of origin).

Since there is a conceptually and statistically significant correlation between the variables which represent the development of a country, the data were analyzed in the following three ways: In accordance with GDP, CPI, and HDI (model A), in accordance with GDP and CPI (model B), and in accordance with CPI and HDI (model C). These models are:

**Model A1:**

*CMR*_it_ ***=*** *α*_i +_ *β*_1_ *×* *Year* *+* *β*_2_ *×* *CSA*_it_ *+* *β*_3_ *× TA*_it_ *+ β*_4_ *× HDI*_it_ *+ β*_5_ *×* *CPI*_it_ *+ β*_6_ *× GDPc*_it_ *+ β*_7_ *× MVC*_it_

Where the country-speciﬁc intercept from the i^th^country is denoted as α_i_s.

**Model A2:**

*CMR*_it_ ***=*** *α*_i +_ *β*_1i_ *×* *Year* *+* *β*_2_ *×* *CSA*_it_ *+* *β*_3_ *× TA*_it_ *+ β*_4_ *× HDI*_it_ *+ β*_5_ *×* *CPI*_it_ *+ β*_6_ *× GDPc*_it_ *+ β*_7_ *× MVC*_it_

where the country-speciﬁc intercept and linear time slope from the i^th^ country are denoted as α_i_ and β_1i_.

**Model B1:**

*CMR*_it_ ***=*** *α*_i +_ *β*_1_ *×* *Year* *+* *β*_2_ *×* *CSA*_it_ *+* *β*_3_ *× TA*_it_ *+ β*_4_ *×* *CPI*_it_ *+ β*_5_ *× GDPc*_it_ *+ β*_6_ *× MVC*_it_

where the country-speciﬁc intercept from the i^th^country is denoted as α_i_s.

**Model B2:**

*CMR*_it_ ***=*** *α*_i +_ *β*_1i_ *×* *Year* *+* *β*_2_ *×* *CSA*_it_ *+* *β*_3_ *× TA*_it_ *+ β*_4_ *×* *CPI*_it_ *+ β*_5_ *× GDPc*_it_ *+ β*_6_ *× MVC*_it_

where the country-speciﬁc intercept and linear time slope from the i^th^ country are denoted as α_i_ and β_1i_.

**Model C1:**

*CMR*_it_ ***=*** *α*_i +_ *β*_1_ *×* *Year* *+* *β*_2_ *×* *CSA*_it_ *+* *β*_3_ *× TA*_it_ *+ β*_4_ *×* *HDI*_it_ *+ β*_5_ *× CPI*_it_ *+ β*_6_ *× MVC*_it_

where the country-speciﬁc intercept from the i^th^country is denoted as α_i_s.

**Model C2:**

*CMR*_it_ ***=*** *α*_i +_ *β*_1i_ *×* *Year* *+* *β*_2_ *×* *CSA*_it_ *+* *β*_3_ *× TA*_it_ *+ β*_4_ *×* *HDI*_it_ *+ β*_5_ *× CPI*_it_ *+ β*_6_ *× MVC*_it_

where the country-speciﬁc intercept and linear time slope from the i^th^ country are denoted as α_i_ and β_1i_.

This paper is the result of a project sponsored by Tehran University of Medical Sciences’ Deputy of Research under Project No. 13517-102-01-90. The funding agent does not have any role in this project except for financial support.

## Results

### Descriptive Results

Data from 56 countries were used in this study. The countries included in this study were: Azerbaijan, Bangladesh, Benin, Bhutan, Bolivia, Burundi, Cambodia, Cameroon, Central African Republic, Comoros, Democratic Republic of Congo, Republic of Congo, Cote d’Ivoire, Djibouti, Arab Republic of Egypt, Eritrea, Gabon, Gambia, Ghana, Guatemala, Haiti, India, Indonesia, Kenya, Kiribati, Kyrgyz Republic, Lao PDR, Lesotho, Madagascar, Maldives, Mauritania, Mongolia, Morocco, Myanmar, Namibia, Nepal, Pakistan, Papua New Guinea, Peru, Rwanda, Sao Tome and Principe, Senegal, Somalia, Sudan, Swaziland, Tajikistan, Tanzania, Timor-Leste, Togo, Turkey, Turkmenistan, Uganda, Uzbekistan, Republic of Yemen, Zambia, and Zimbabwe.

**Figure 1:**
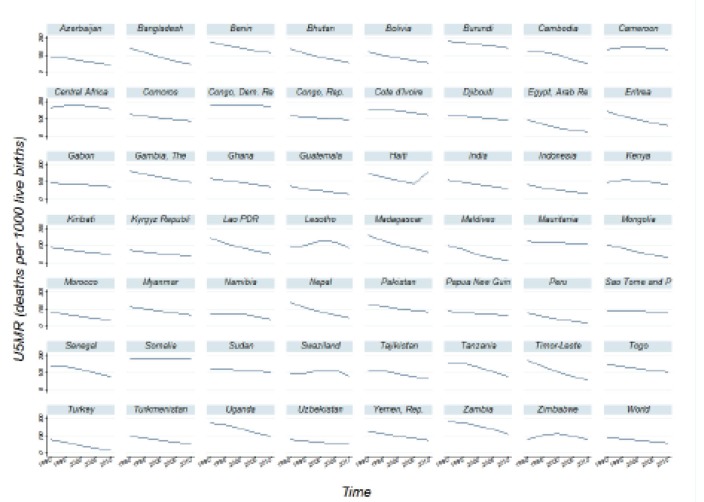
Country-Specific Trends of U5MR Over a 20-Year Period From 1990 to 2010. Abbreviation: U5MR, under-five mortality rate.

Figure 1 summarizes the U5MR data for the studied countries in the relevant period. Except for Haiti, Rwanda, Lesotho, Swaziland, and Zimbabwe, the U5MR reduction trend over time appears to be linear for all other countries. Some countries such as Maldives, Turkey, Arab Republic of Egypt, and Peru have experienced a decrease in the rate of U5MR up to 75% compared to their U5MR in 1990. The relative reduction in the rate of U5MR in other countries such as the Democratic Republic of Congo, Lesotho, and Central African Republic, and Cameroon was less than 10% for this period. U5MR was constant for some countries such as Somalia. Some other countries such as Zimbabwe and Haiti have even experienced a 10% increase in their U5MR.

**Figure 2:**
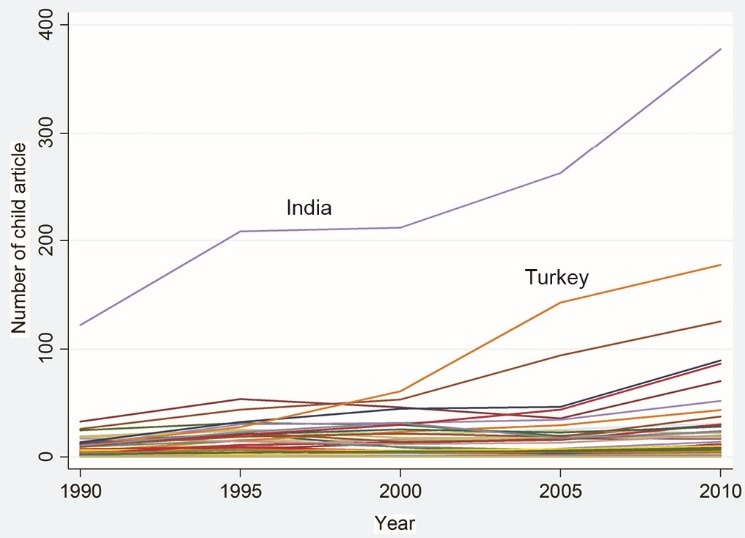
Country-Specific Trends of CSA Over a 20-Year Period From 1990 to 2020. Abbreviation: CSA, child-specific articles.

**Figure 3:**
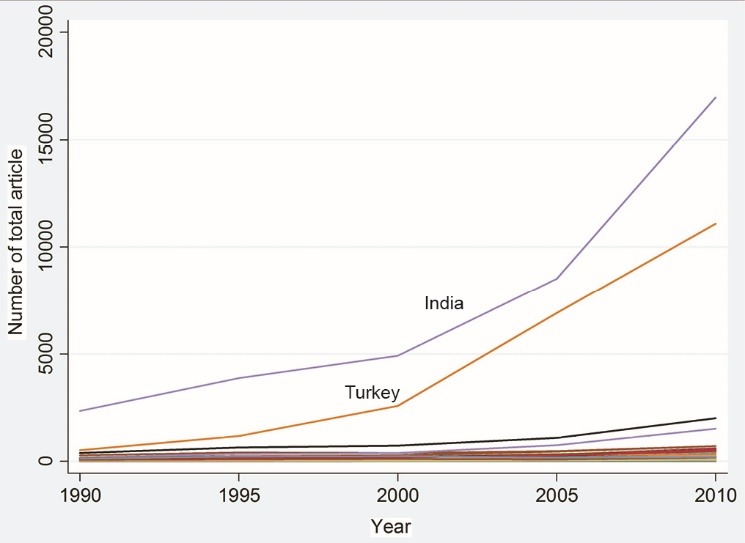
Country-Specific Trends of TA Over a 20-Year Period From 1990 to 2020. Abbreviation: TA, total articles.

The main independent variables in this study were the TA and number of CSA. Figures 2 and 3 show the changes in these variables during the periods under study, respectively. As illustrated, except for India and Turkey, these two indicators have not changed much over time.

### Univariate Analysis

Table 2 shows the univariate analysis between the dependent variable (U5MR) and the independent variables. As shown in this table, all independent variables in the univariate analysis have a significant relationship with U5MR. Table 3 shows that these variables significantly correlate with each other. There is also a significant correlation between HDI and GDPc for the countries under study, and the TA published in a country and the number of CSA in the same country.

**Table 2:** The Relationship Between U5MR and the Independent Variables (Univariate Analysis)

**Covariate**	**Coefficients**	**SE**	**Z**	***P*** ** Value**
CSA	-0.43	0.09	-4.98	< .0001
TA	-0.01	0.00	-3.80	< .0001
HDI	-408.43	17.14	-23.82	< .0001
GDPc	-0.03	0.00	-7.15	< .0001
MVC	-1.21	0.09	-13.72	< .0001
CPI	-16.96	5.87	-2.89	< .0001
Time	-2.36	0.11	-20.54	< .0001

Abbreviations: CSA, child-specific articles; TA, total articles; HDI, human development index; U5MR, under-five mortality rate; GDPc: gross domestic production per capita (constant US$2000); MVC, measles vaccination coverage; CPI, corruption perception index; SE, standard error.

**Table 3:** Pearson Correlation Coefficients Between the Independent Variables and U5MR for 56 Countries Between 1990 and 2010

** **	**HDI**	**U5MR**	**CSA**	** TA**	**GDPc**	**MVC**	**CPI**	**Time**
** HDI **	1.00							
** U5MR **	-0.83	1.00						
**CSA **	0.06	-0.13	1.00					
** TA **	0.18	-0.21	0.89	1.00				
** GDPc **	0.67	-0.49	0.05	0.22	1.00			
** MVC **	0.51	-0.55	-0.01	0.05	0.21	1.00		
** CPI **	0.40	-0.36	0.04	0.15	0.47	0.30	1.00	
** Time **	0.32	-0.44	0.14	0.14	0.08	0.43	0.00	1.00

Abbreviations: CSA, child-specific articles; TA, total articles; HDI, human development index; U5MR, under-five mortality rate; GDPc: gross domestic production per capita (constant US$2000); MVC, measles vaccination coverage; CPI, corruption perception index.

### Multivariate Analysis

Among all six models, ‘the random intercept and random slope models’ has lower residuals and thereby functions better than ‘the random intercept models.’ As shown in Tables 4, 5, and 6, in each of the A, B, and C models, the significant variables are identical to each other in ‘the random intercept models’ and ‘random intercept and random slope models.’

**Table 4:** Coefficients and Features Of Models A (GDP, CPI, and HDI Variables as the Indicators of Development)

	**Coefficient**	**SE**	**z**	***P*** ** > |z|**	**95% CI**	**Standard Coefficient**
Model A1							
CSA	-0.17	0.09	-1.84	.066	-0.35	0.01	-4.78
TA	0.00	0.00	1.50	.135	0.00	0.01	3.11
HDI	-299.96	27.84	-10.77	.000	-354.52	-245.39	-31.88
CPI	-5.22	4.21	-1.24	.215	- 13.46	3.02	-3.83
GDPc	0.00	0.00	0.86	.392	0.00	0.01	2.60
MVC	-0.11	0.09	-1.23	.218	-0.29	0.07	-2.09
Time	-0.80	0.16	-5.02	.000	-1.12	-0.49	-5.68
Cons	264.69	14.28	18.54	.000	236.70	292.67	98.95
Residual	10.13						
Model A2							
CSA	-0.17	0.09	-1.94	.053	-0.33	0.00	-4.67
TA	0.00	0.00	1.45	.148	0.00	0.01	2.87
HDI	-296.15	28.88	-10.25	.000	-352.76	-239.55	-31.48
CPI	-6.20	4.23	-1.47	.143	-14.49	2.09	-4.55
GDPc	0.00	0.00	1.37	.171	0.00	0.01	4.25
MVC	-0.08	0.08	-0.99	.320	-0.25	0.08	-1.52
Time	-0.88	0.21	-4.23	.000	-1.28	-0.47	-6.21
Cons	262.65	14.51	18.10	.000	234.20	291.10	98.99
Residual	7.30						

Abbreviations: CSA, child-specific articles; TA, total articles; HDI, human development index; U5MR, under-five mortality rate; GDPc: gross domestic production per capita (constant US$2000); MVC, measles vaccination coverage; CPI, corruption perception index; SE, standard error.

**Table 5:** Coefficients and Features of Model B (GDP, CPI Variables as the Indicators of Development)

	**Coefficient**	**SE**	**z**	***P*** ** >|z|**	**95% CI**	**Standard Coefficient**
Model B1							
CSA	-0.21	0.11	-1.87	.061	-0.43	0.01	-5.89
TA	0.01	0.00	1.77	.077	0.00	0.01	4.4
CPI	-4.84	5.45	-0.89	.374	-15.52	5.83	-3.55
GDPc	-0.02	0.00	-4.64	.000	-0.02	-0.01	-13.97
MVC	-0.46	0.10	-4.78	.000	-0.64	-0.27	-8.41
Time	-1.59	0.16	-9.64	.000	-1.91	-1.27	-11.25
Cons	171.32	15.20	11.27	.000	141.53	201.12	98.62
Residual	12.08						
Model B2							
CSA	-0.13	0.10	-1.31	.190	-0.32	0.06	-3.66
TA	0.00	0.00	1.47	.142	0.00	0.01	3.25
CPI	-6.87	5.60	-1.23	.220	-17.85	4.11	-5.04
GDPc	-0.01	0.00	-3.10	.002	-0.02	0.00	-9.89
MVC	-0.28	0.08	-3.52	.000	-0.44	-0.12	-5.16
Time	-1.86	0.22	-8.55	.000	-2.28	-1.43	-13.16
Cons	163.45	15.40	10.61	.000	133.27	193.64	98.54
Residual	7.84						

Abbreviations: CSA, child-specific articles; TA, total articles; HDI, human development index; U5MR, under-five mortality rate; GDPc: gross domestic production per capita (constant US$2000); MVC, measles vaccination coverage; CPI, corruption perception index; SE, standard error.

**Table 6:** Coefficients and Features of Model C (CPI and HDI Variables as the Indicators of Development)

	**Coefficient**	**SE**	**z**	***P*** ** >|z|**	**95% CI**	**Standard Coefficient**
Model C1							
CSA	-0.18	0.09	-2.02	.043	-0.36	-0.01	-5.16
TA	0.00	0.00	1.76	.078	0.00	0.01	3.49
HDI	-278.11	23.36	-11.91	.000	-323.90	-232.33	-29.56
CPI	-3.11	4.02	-0.77	.439	-10.99	4.76	-2.28
MVC	-0.12	0.09	-1.40	.161	-0.30	0.05	-2.27
Time	-0.82	0.16	-5.19	.000	-1.13	-0.51	-5.88
Cons	251.44	12.74	19.74	.000	226.48	276.41	98.14
Residual	10.13						
Model C2							
CSA	-0.17	0.09	-2.01	.045	-0.35	0.00	-4.92
TA	0.00	0.00	1.74	.082	0.00	0.01	3.42
HDI	-267.46	24.19	-11.06	.000	-314.87	-220.05	-28.43
CPI	-3.71	4.01	-0.93	.355	-11.57	4.15	-2.72
MVC	-0.13	0.08	-1.60	.111	-0.29	0.03	-2.37
Time	-0.89	0.20	-4.53	.000	-1.28	-0.51	-6.37
Cons	249.31	12.88	19.35	.000	224.07	274.56	98.29
Residual	7.73						

Abbreviations: CSA, child-specific articles; TA, total articles; HDI, human development index; U5MR, under-five mortality rate; GDPc: gross domestic production per capita (constant US$2000); MVC, measles vaccination coverage; CPI, corruption perception index; SE, standard error.

Model A2, B2, and C2 have similar residuals but in the A2 and C2 models (both of which have HDI as the development index), the time coefficients are both similar to each other and they are lower than the time coefficient in model B2. This shows that models A2 and C2 are more appropriate. In the A2 and C2 models, the same variables of CSA, HDI, and time are significant. In the A2 and C2 models, the coefficient of this variable was estimated at -0.17 (*P* = .05 and .045).

Among the random intercept models (A1, B1, and C1), A1 and C1 are similar to each other in terms of residuals and act better than B1.

In the A1 and C1 models, the same variables of HDI and time are significant, in C1 the CSA is significant, and in A1 CSA is near significant (*P* = .066).

In general, between the two variables of TA and CSA, CSA is significant or near significant in all the models.

## Discussion

The purpose of this study was to evaluate the impact of health research on the reduction of U5MR through the ecologic approach. The three models used in the ‘random intercept and slope models’ created a better fit in terms of residuals. In the best models, A2 and C2, the coefficients of CSA were significant or near significant (*P* = .053 in A2 and .045 in C2). This means that with the addition of every 100 CSA, the rate of U5MR decreased by 17 per 1000 live births. Because the coefficient of HDI was higher than the other variables, it should be considered when planning for the reduction of U5MR. As mentioned in the 2015 MDGs report, more work should be done for reducing child mortality and human development should be at the center of this focus. However, it must be noted that this study is an ecologic one, and that there are certain limitations in the interpretation of its findings, and that confounding variables must be considered in its interpretation. For example, it is not clear how much the impact of research has been in that country, and whether or not the numbers of articles are associated with a high impact rate.

Among the countries examined in this study, no significant changes in CSA were observed over time. One reason behind the lack of a strong relationship between CSA and U5MR maybe the lack of variance in the CSA.

The association between health research and improvement of health status has been investigated in a limited number of studies. In one study, the relationship between the number of publications in PubMed (as a proxy of stoke knowledge) and cancer mortality was examined in the United States. There, they observed a strong inverse relationship between the number of publications which had received research funding and cancer mortality upon considering a 5 and 10 years’ lag. The latter finding confirms the 5 years lag time used in our study. In other ecological studies, the final goal has been the economic payback of research budget.^14-19^ For example, an Australian study showed that returns to investment in cardiovascular diseases were nearly 8 times, and in respiratory diseases they were 6 times the annual R&D investment.^14^ Furthermore, a British study indicated that the best-estimate internal rate of return was 10% in cancer-related diseases^19^ and 9% in cardiovascular diseases research.^17^ In order to nullify the attribution in these ecological studies, based on certain presumptions, the level of attribution was set at 50% (analysis was repeated twice with 30% and 70%).^17^ Anyhow, the attribution problem is an important consideration in evaluation studies, although some references have suggested using contribution instead of attribution, which is a less rigorous and quantitative definition.^20^ In the present study, efforts have been made to introduce a linear model to solve the attribution problem‏. Moreover, the variables relevant to health improvement outside the health system were considered in estimating the impact of research on health improvement. In these models, the effect of HDI was approximately 5.78 to 6.74 times more than the CSA. Now, the question that arises is whether this amount is plausible or not?

The point to consider here is that in most of these models, CSA is either significant or near significant, while MVC is not so. Studies have shown that an MVC <59% is not significantly associated with measles-related deaths.^21^ In the current study, the MVC of the years under study was 60%–79% in 30% of the countries, and <59% in 66% of the countries, so the result is not beyond expectation.

The aforementioned models’ residuals indicate that the models for which HDI is the proxy of country development function better. This confirms that HDI represents country development in terms of health, better than does GDP. In all the models, the time variable was significant. This is because variables that are time-dependent and influence U5MR are not included in the models. Moreover, when HDI exists in a given model, the health system-related indexes (ie, MVC) lose their significance. This in turn means that HDI is a valid variable in describing the status of development for countries. There is no consensus on an index appropriate for measuring the development of countries. Starting from 1954 and for many consecutive years, GDP was considered as an indicator of social welfare. This indicator, however, has always been questioned because of two important reasons: It ignores the distribution of economic growth in the society and it considers economic welfare as the only factor of welfare. As a result, the HDI was proposed by the UNDP for this purpose and is annually calculated for countries since 1990.^22^ As evident in this study, unlike GDP, if HDI is included in a model, the contribution of time is reduced which shows a more proper relationship between HDI and country development. These results are in favor of the demand for health (Grossman) model, as, by accepting the notion that the HDI is an acceptable indicator of a country’s economic, social, and environmental status, we observe a strong association between this index and health (U5MR reduction).

## Limitations

The evaluation of research production and health impact by ecological methods is very young, thus, knowing the limitations will help future studies to picture the reality more. There were certain limitations in this study.

We only used the number of articles published by the countries as the proxy of health research systems, which is not an appropriate index and causes several imitations in the interpretation of results in certain ways. Firstly, the number of articles does not reflect the quality of research which is an important factor for its implementation. Thence, we suggest using some indicators as the proxy of quality of research, such as citation analysis and journal impact factor^23-25^ alongside the number of papers in future studies. Secondly, research studies are implemented only when the necessary policies, programs, and resources are available and only when the effects of their implementation become apparent.^26^ Consequently, other effects such as the exchange between knowledge generating and using organizations and whether the results of research studies are made available to the policy-makers at the right time, with the right language, and through the right channel should also be considered. Thirdly, international studies that have played a role in reducing U5MR in specific country, but have not been counted in that country’s articles; there is no solution for this problem, as it cannot be specified which study has affected which country. However, as a confounder, the TA around the world can be entered into the model (it would be better to enter the articles of those countries that have not been included in the study, and better still if the types of study could be included as well).

Fourthly, we did not investigate the impact of different types of studies on health research. Obviously, not all types of health research have the same bearing on the improvement of health. For example, studies which evaluate the effectiveness of interventions in special contexts or those that investigate the implementation of those interventions are more effective in improving health.^27^ As a result, an increase in the total number of studies will not have any considerable impact on the improvement of health. Paying attention to the distribution of different types of studies is crucial for an accurate evaluation of the impact of health research. Perhaps if the impact of health system, health services or universal health coverage researches in developing countries were reviewed, the impact would be different.

Another limitation is using one indicator as the proxy of health system performance and health research system. We suggest using more than one indicator for them in future studies in order to better picture reality. As a proxy of health system performance, the studies can use indicators which have been introduced by the WHO.^28^

## Conclusion

Child-related research has a significant impact on U5MR reduction. However, this contribution is negligible compared to the effect of development of countries and other time-related factors. Unlike GDP, if HDI is included in a model, the contribution of time reduces which implies the existence of a stronger relationship between HDI and health improvement.

Through this study, a new question arises in the context of research policy-making: “What is the appropriate impact level of the health research system on health improvement?”

In order to gain deeper understanding of the contribution of countries’ health research systems toward the reduction of U5MR, conducting studies that investigate the impact of different types of research is warranted. Moreover, the effect of research will be examined with greater transparency if the ‘exchange’ variable is also included in the model in future studies.

## Ethical issues

This study was approved by the Institutional Review Board of University of Tehran’s Medical Science Faculty which follows the Helsinki Declaration.

## Competing interests

Authors declare that they have no competing interests.

## Authors’ contributions

RM has contributed to design and discussion of this paper. BY has contributed to literature review, design, drafting the paper, and discussion. MP has contributed to analysis of data. SN has contributed to data collection.

## Authors’ affiliations

^1^Knowledge Utilization Research Center, Tehran University of Medical Sciences, Tehran, Iran. ^2^Epidemiology and Biostatistics Department, School of Public Health, Tehran University of Medical Sciences, Tehran, Iran.

## Supplementary files

Supplementary file 1 contains Appendix 1 (Search strategy).

## Key messages

**Implications for policy makers**

- With the addition of every 100 child-specific articles (CSA) the rate of under-five mortality rate (U5MR) decreased by 17 per 1000 live births.
- Through this study, a new question arises in the context of research policy-making: “What is the appropriate impact level of the health research system on health improvement?”

**Implications for public**

Research on child mortality can help reduce it by offering useful evidence for utilization in policy-making.
