# Supplementary material for: Impact of Health Research Systems on Under-5 Mortality Rate: A Trend Analysis
Source: Int J Health Policy Manag. 2016 Nov 26;6(7):395–402. doi: 10.15171/ijhpm.2016.147 (PMC5505109; doi:10.15171/ijhpm.2016.147)
Supplement: Supplementary file 1 — contains Appendix 1 (Search strategy). [file ijhpm-6-395-s001.pdf]

IJHPM
International Journal of Health Policy and Management

Appendix 1: Search strategy

Search strategy: Total publication by country

Kyrgyzstan

Kyrgyzstan [title/abstract] OR Kyrgyzstan [AD] OR Kyrgyzstan [Mesh] OR "Kyrgyz Republic" [title/abstract] OR "Kyrgyz Republic" [AD] OR "Kyrgyz Republic" [Mesh] OR Bishkek [title/abstract] OR Bishkek [AD]

Kyrgyzstan [ti] OR Kyrgyzstan [AD] OR Kyrgyzstan [Mesh] OR "Kyrgyz Republic" [ti] OR "Kyrgyz Republic" [AD] OR "Kyrgyz Republic" [Mesh] OR Bishkek [ti] OR Bishkek [AD]

Guatemala

Guatemala [ti] OR Guatemala [AD] OR Guatemala [Mesh]

Guatemala [title/abstract] OR Guatemala [AD] OR Guatemala [Mesh]

Peru

(peru* [ti] NOT perugia [ti]) OR peru [AD] OR peru [Mesh] OR lima [ti] OR lima [AD]

(peru* [title/abstract] NOT perugia [title/abstract]) OR peru [AD] OR peru [Mesh] OR lima [title/abstract] OR lima [AD]

Zimbabwe

Zimbabwe [ti] OR Zimbabwe[AD] OR Zimbabwe[Mesh] OR Harare[ti] OR Harare[AD]

Zimbabwe [title/abstract] OR Zimbabwe[AD] OR Zimbabwe[Mesh] OR Harare[title/abstract] OR Harare[AD]

Turkey

turkey [ti] OR turkey [AD] OR turkey [Mesh] OR ankara [ti] OR ankara [AD] OR istanbul[ti] OR istanbul[AD]

turkey [title/abstract] OR turkey [AD] OR turkey [Mesh] OR ankara [title/abstract] OR ankara [AD] OR istanbul[title/abstract] OR istanbul[AD]

Indonesia

Indonesia [ti] OR Indonesia [AD] OR Indonesia [Mesh] OR Jakarta[ti] OR Jakarta[AD]

Indonesia [title/abstract] OR Indonesia [AD] OR Indonesia [Mesh] OR Jakarta[title/abstract] OR Jakarta[AD]

Kiribati

Kiribati [ti] OR kiribati [ad] OR kiribati [Mesh] OR Tarawa [ti] OR Tarawa [ad]

Kiribati [title/abstract] OR kiribati [ad] OR kiribati [Mesh] OR Tarawa [title/abstract] OR Tarawa [ad]

Morocco

Morocco [ti] OR morocco [ad] OR morocco [mesh] OR rabat[ti] OR rabat[ad]

morocco [title/abstract] OR morocco [ad] OR morocco [mesh] OR rabat[title/abstract] OR rabat[ad]

Egypt

egypt* [ti] OR egypt [ad] OR egypt [mesh] OR cairo [ti] OR cairo[ad]

egypt* [title/abstract] OR egypt [ad] OR egypt [mesh] OR cairo [title/abstract] OR cairo[ad]

Papua New Guinea

"Papua New Guinea" [ti] OR "Papua New Guinea" [ad] OR "Papua New Guinea" [mesh] OR "Port Moresby" [ti] OR "Port Moresby"[ad]

"Papua New Guinea" [title/abstract] OR "Papua New Guinea" [ad] OR "Papua New Guinea" [mesh] OR "Port Moresby" [title/abstract] OR "Port Moresby"[ad]

Swaziland

swaziland [ti] OR swaziland [ad] OR swaziland [mesh] OR Mbabane[ti] OR Mbabane[ad] OR lobamba[ti] OR lobamba[ad]

swaziland [title/abstract] OR swaziland [ad] OR swaziland [mesh] OR Mbabane[title/abstract] OR Mbabane[ad] OR lobamba[title/abstract] OR lobamba[ad]

Lesotho

lesotho [ti] OR lesotho [ad] OR lesotho [mesh] OR Maseru[ti] OR Maseru[ad]

lesotho [title/abstract] OR lesotho [ad] OR lesotho [mesh] OR Maseru[title/abstract] OR Maseru[ad]

Gabon

gabon [ti] OR gabon[ad] OR gabon [mesh] OR Libreville[ti] OR Libreville[ad]

gabon [title/abstract] OR gabon[ad] OR gabon [mesh] OR Libreville[title/abstract] OR Libreville[ad]

Sao Tome and Principe

"Sao Tome" [ti] OR "Sao Tome" [ad] OR "Sao Tome"[mesh]

"Sao Tome" [title/abstract] OR "Sao Tome" [ad] OR "Sao Tome"[mesh]

Azerbaijan

(Azerbaijan [ti] OR Azerbaijan [ad] OR Azerbaijan [mesh] OR baku[ti] OR baku[ad]) NOT (IRAN [title/abstract] OR iran [ad] OR iran [mesh] OR baku*[au])

(Azerbaijan [title/abstract] OR Azerbaijan [ad] OR Azerbaijan [mesh] OR baku[title/abstract] OR baku[ad]) NOT (IRAN [title] OR iran [ad] OR iran [mesh] OR baku*[au])

Turkmenistan

Turkmenistan [ti] OR Turkmenistan[ad] OR Turkmenistan[mesh] OR Ashgabat[ti] OR Ashgabat[ad]

Turkmenistan [title/abstract] OR Turkmenistan[ad] OR Turkmenistan[mesh] OR Ashgabat[title/abstract] OR Ashgabat[ad]

Kenya
kenya[ti] OR kenya[ad] OR kenya[mesh] OR Nairobi[ti] OR Nairobi[ad]
kenya[title/abstract] OR kenya[ad] OR kenya[mesh] OR Nairobi[title/abstract] OR Nairobi[ad]
Mongolia
(mongolia[ti] OR mongolia[ad] OR mongolia[mesh] OR Ulaanbaatar[ti] OR Ulaanbaatar[ad]) NOT “mongolian spot”
(mongolia[title/abstract] OR mongolia[ad] OR mongolia[mesh] OR Ulaanbaatar[title/abstract] OR Ulaanbaatar[ad]) NOT “mongolian spot”
Congo,rep.
(congo[ti] OR congo[ad] OR congo[mesh] OR Brazzaville[ti] OR Brazzaville[ad]) NOT (“congo red” OR Democratic OR DRC OR Zaire OR kinshasa OR crimean)
(congo[title/abstract] OR congo[ad] OR congo[mesh] OR Brazzaville[title/abstract] OR Brazzaville[ad]) NOT (“congo red” OR Democratic OR DRC OR Zaire OR kinshasa OR crimean)
Maldives
maldives [ti] OR maldives[ad]
maldives [title/abstract] OR maldives[ad]
Cambodia
Cambodia[ti] OR Cambodia[ad] OR Cambodia[mesh] OR “Phnom Penh” [ti] OR “Phnom Penh” [ad]
Cambodia[title/abstract] OR Cambodia[ad] OR Cambodia[mesh] OR “Phnom Penh” [title/abstract] OR “Phnom Penh” [ad]
Tajikistan
Tajikistan[ti] OR Tajikistan[ad] OR Tajikistan[mesh] OR Dushanbe [ti] OR Dushanbe [ad]
Tajikistan[title/abstract] OR Tajikistan[ad] OR Tajikistan[mesh] OR Dushanbe [title/abstract] OR Dushanbe [ad]
Myanmar
Myanmar[ti] OR Myanmar[ad] OR Myanmar[mesh] OR Naypyidaw [ti] OR Naypyidaw [ad]
Myanmar[title/abstract] OR Myanmar[ad] OR Myanmar[mesh] OR Naypyidaw [title/abstract] OR Naypyidaw [ad]
India
(India[ti] OR India[ad] OR india[mesh] OR “New Delhi”[ti] OR “New Delhi”[ad]) NOT (“indian ocean” OR “indian ink” OR “Indian american” OR “american indian” OR “indian childhood cirrhosis”)
(India[tile/abstract] OR India[ad] OR india[mesh] OR “New Delhi”[title/abstract] OR “New Delhi”[ad]) NOT (“indian ocean” OR “indian ink” OR “Indian american” OR “american indian” OR “indian childhood cirrhosis”)
Ghana
ghana[ti] OR ghana[ad] OR ghana[mesh] OR Accra[ti] OR Accra[ad]
ghana[title/abstract] OR ghana[ad] OR ghana[mesh] OR Accra[title/abstract] OR Accra[ad]
Bolivia
bolivia[ti] OR bolivia[ad] OR bolivia[mesh] OR “La Paz”[ti] OR “ La Paz”[ad]
bolivia[title/abstract] OR bolivia[ad] OR bolivia[mesh] OR “La Paz”[title/abstract] OR “ La Paz”[ad]
Djibouti
Djibouti[ti] OR Djibouti[ad] OR Djibouti[mesh]
Djibouti[title/abstract] OR Djibouti[ad] OR Djibouti[mesh]
Sudan
(sudan[ti] or sudan[ad] or sudan[mesh] or khartoum [ti] or khartoum [ad]) not (sudanophilic or sudan* [au] or “sudan stain” OR “sudan black”)
(sudan[title/abstract] OR sudan[ad] OR sudan[mesh] OR Khartoum [title/abstract] OR Khartoum [ad]) NOT (sudanophilic OR sudan* [au] OR “sudan stain” OR “sudan black”)
Yemen
Yemen[ti] OR Yemen[ad] OR Yemen[mesh] OR Sana’a[ti] OR Sana’a[ad]
Yemen[title/abstract] OR Yemen[ad] OR Yemen[mesh] OR Sana’a[title/abstract] OR Sana’a[ad]
Comoros
(comoros[ti] or comoros[ad] or comoros[mesh] or Moroni[ti] or Moroni[ad]) not (moroni[au] OR comor* [au])
(comoros[title/abstract] or comoros[ad] or comoros[mesh] or Moroni[title/abstract] or Moroni[ad]) not (moroni[au] OR comor* [au])
Mauritania
Mauritania[ti] or Mauritania[ad] or Mauritania[mesh] or Nouakchott[ti] or Nouakchott[ad]
Mauritania[title/abstract] or Mauritania[ad] or Mauritania[mesh] or Nouakchott[title/abstract] or Nouakchott[ad]
Pakistan
(pakistan[ti] or pakistan[ad] or pakistan[mesh] OR Islamabad[ti] or Islamabad[ad] OR karachi[ti] or karachi[ad] OR Lahore[ti] OR Lahore[ad]) NOT islamabad*[au]
(pakistan[title/abstract] or pakistan[ad] or pakistan[mesh] OR Islamabad[title/abstract] or Islamabad[ad] OR karachi[title/abstract] or karachi[ad] OR Lahore[title/abstract] OR Lahore[ad]) NOT islamabad*[au]
Nepal
Nepal[ti] or Nepal[ad] or Nepal[mesh] OR Kathmandu[ti] or Kathmandu[ad]
Nepal[title/abstract] or Nepal[ad] or Nepal[mesh] OR Kathmandu[title/abstract] or Kathmandu[ad]
Bangladesh
Bangladesh[ti] or Bangladesh[ad] OR Bangladesh[mesh] OR Dhaka[ti] OR Dhaka[ad]
Bangladesh[title/abstract] or Bangladesh[ad] OR Bangladesh[mesh] OR Dhaka[title/abstract] OR Dhaka[ad]
Cameroon
Cameroon[ti] or Cameroon[ad] OR Cameroon[mesh] OR Yaounde[ti] OR Yaounde[ad]
Cameroon[title/abstract] or Cameroon[ad] OR Cameroon[mesh] OR Yaounde[title/abstract] OR Yaounde[ad]
Bhutan
Bhutan[ti] OR Bhutan[ad] OR Bhutan[mesh] OR Thimphu[ti] OR Thimphu[ad]

Bhutan[title/abstract] OR Bhutan[ad] OR Bhutan[mesh] OR Thimphu[title/abstract] OR Thimphu[ad]

Eritrea

(Eritrea*[ti] OR Eritrea[ad] OR Eritrea[mesh] OR Asmara[ti] OR Asmara[ad]) NOT Asmara*[au]

(Eritrea*[title/abstract] OR Eritrea[ad] OR Eritrea[mesh] OR Asmara[title/abstract] OR Asmara[ad]) NOT Asmara*[au]

Togo

(Togo[ti] OR togolese[ti] OR togo[ad] OR togo[mesh] OR Lome[ti] OR Lome[ad]) NOT (togo*[au] OR Lome*[au])

(Togo[title/abstract] OR togolese[title/abstract] OR togo[ad] OR togo[mesh] OR Lome[title/abstract] OR Lome[ad]) NOT (togo*[au] OR Lome*[au])

Senegal

Senegal[ti] OR Senegal[ad] OR Senegal[mesh] OR Dakar[ti] OR Dakar[ad]

Senegal[title/abstract] OR Senegal[ad] OR Senegal[mesh] OR Dakar[title/abstract] OR Dakar[ad]

Haiti

Haiti[ti] OR Haiti[ad] OR Haiti[mesh] OR Port-au-Prince[ti] OR Port-au-Prince[ad] OR «Port au Prince»[ti] OR «Port au Prince»[ad]

Haiti[title/abstractt] OR Haiti[ad] OR Haiti[mesh] OR Port-au-Prince[title/abstract] OR Port-au-Prince[ad] OR «Port au Prince»[title/abstract] OR «Port au Prince»[ad]

Cote d'Ivoire(Ivory coast)

"Cote d'Ivoire"[ti] OR "Ivory coast" [ti] OR "Cote d'Ivoire"[ad] OR "ivory coast"[ad] OR "Cote d'Ivoire"[mesh] OR "Ivory coast"[Mesh] OR Abidjan[ti] OR Abidjan[ad]

"Cote d'Ivoire"[title/abstract] OR "Ivory coast" [title/abstract] OR "Cote d'Ivoire"[ad] OR "ivory coast"[ad] OR "Cote d'Ivoire"[mesh] OR "Ivory coast"[Mesh] OR Abidjan[title/abstract] OR Abidjan[ad]

Gambia

Gambia[ti] OR Gambia[ad] OR Gambia[Mesh] OR Banjul[ti] OR Banjul[ad]

Gambia[title/abstract] OR Gambia[ad] OR Gambia[Mesh] OR Banjul[title/abstract] OR Banjul[ad]

Lao PDR(Laos)

("Lao PDR" [ti] OR Laos[ti] OR "Lao PDR"[ad] OR Laos[ad] "Lao PDR"[Mesh] OR Laos[Mesh] OR Vientiane[ti] OR Vientiane[ad]) NOT laos*[au]

("Lao PDR" [title/abstract] OR Laos[title/abstract] OR "Lao PDR"[ad] OR Laos[ad] "Lao PDR"[Mesh] OR Laos[Mesh] OR Vientiane[title/abstract] OR Vientiane[ad]) NOT laos*[au]

Tanzania

Tanzania [ti] OR Tanzania[ad] OR Tanzania[Mesh] OR "Dar es Salaam"[ti] OR "Dar es Salaam" [ad] OR "Dar-es-Salaam"[ti] OR "Dar-es-Salaam" [ad]

Tanzania [title/abstract] OR Tanzania[ad] OR Tanzania[Mesh] OR "Dar es Salaam"[title/abstract] OR "Dar es Salaam" [ad] OR "Dar-es-Salaam"[title/abstract] OR "Dar-es-Salaam" [ad]

Madagascar

Madagascar[ti] OR Madagascar[ad] OR Madagascar[Mesh] OR Antananarivo[ti] OR Antananarivo [ad]

Madagascar[title/abstract] OR Madagascar[ad] OR Madagascar[Mesh] OR Antananarivo[title/abstract] OR Antananarivo [ad]

Rwanda

Rwanda[ti] OR Rwanda[ad] OR Rwanda[Mesh] OR Kigali[ti] OR Kigali[ad]

Rwanda[title/abstract] OR Rwanda[ad] OR Rwanda[Mesh] OR Kigali[title/abstract] OR Kigali[ad]

Zambia

ZAMBIA[Ti] OR Zambia[ad] OR Zambia[Mesh] OR Lusaka [ti] OR Lusaka[ad]

ZAMBIA[Title/abstract] OR Zambia[ad] OR Zambia[Mesh] OR Lusaka [title/abstract] OR Lusaka[ad]

Somalia

somalia[TI] OR somalia[ad] OR somalia[Mesh] OR Mogadishu[ti] OR Mogadishu[ad]

somalia[Title/abstract] OR somalia[ad] OR somalia[Mesh] OR Mogadishu[title/abstract] OR Mogadishu[ad]

Timor-Leste

(Timor-Leste[TI] OR Timor-Leste[ad] OR Timor-Leste[Mesh] OR "Timor Leste"[TI] OR "Timor Leste"[ad] OR "Timor Leste"[Mesh] OR "East Timor"[ti] OR "east timor"[ad] OR "east timor"[Mesh] OR Dili[ti] OR Dili[ad]) NOT Dili*[au]

(Timor-Leste[Title/abstract] OR Timor-Leste[ad] OR Timor-Leste[Mesh] OR "Timor Leste"[Title/abstract] OR "Timor Leste"[ad] OR "Timor Leste"[Mesh] OR "East Timor"[title/abstract] OR "east timor"[ad] OR "east timor"[Mesh] OR Dili[title/abstract] OR Dili[ad]) NOT Dili*[au]

Uganda

Uganda[TI] OR Uganda[ad] OR Uganda[Mesh] OR Kampala[ti] OR Kampala[ad]

Uganda[Title/abstract] OR Uganda[ad] OR Uganda[Mesh] OR Kampala[title/abstract] OR Kampala[ad]

Benin

(Benin[TI] OR Benin[ad] OR Benin[Mesh] OR Porto-Novo[ti] OR Porto-Novo[ad]) NOT (Benin*[au] OR "benin city")

(Benin[Title/abstract] OR Benin[ad] OR Benin[Mesh] OR Porto-Novo[title/abstract] OR Porto-Novo[ad]) NOT (Benin*[au] OR "benin city")

Burundi

(Burundi[TI] OR Burundi[ad] OR Burundi[Mesh] OR Bujumbura[ti] OR Bujumbura[ad]) NOT Burundi*[au]

(Burundi[Title/abstract] OR Burundi[ad] OR Burundi[Mesh] OR Bujumbura[title/abstract] OR Bujumbura[ad]) NOT Burundi*[au]

Central African republic

"central african republic" [ti] OR "central african republic"[AD] OR "central african republic" [Mesh] OR Bangui [ti] OR Bangui [AD]

"central african republic" [Title/abstract] OR "central african republic"[AD] OR "central african republic" [Mesh] OR Bangui [Title/abstract] OR Bangui [AD]

Democratic Republic of Congo

(("democratic republic" [ti] AND congo [ti]) OR ("democratic republic" [ad] AND congo [ad]) OR "Democratic Republic of

the Congo" [mesh] OR Kinshasa[ti] OR Kinshasa[AD] OR Zaire[ti] OR Zaire[ad]) NOT ("congo red" OR Crimean OR Brazzaville)
(("Democratic republic" [Title/abstract] AND Congo [Title/abstract]) OR ("democratic republic" [ad] AND congo [ad]) OR "Democratic Republic of the Congo" [mesh] OR Kinshasa[Title/abstract] OR Kinshasa[AD] OR Zaire[ti] OR Zaire[ad]) NOT ("congo red" OR Crimean OR Brazzaville)

Namibia

Namibia[TI] OR Namibia[ad] OR Namibia[Mesh] OR Windhoek [ti] OR Windhoek [ad]
Namibia[Title/abstract] OR Namibia[ad] OR Namibia[Mesh] OR Windhoek [Title/abstract] OR Windhoek [ad]

Uzbekistan

Uzbekistan[TI] OR Uzbekistan[ad] OR Uzbekistan[Mesh] OR Tashkent [ti] OR Tashkent [ad]
Uzbekistan[Title/abstract] OR Uzbekistan[ad] OR Uzbekistan[Mesh] OR Tashkent [Title/abstract] OR Tashkent [ad]

Search strategy: Child-specific search strategy

Child Keywords

"Child"[Mesh] OR "Child Health Services"[Mesh] OR "Child Nutrition Sciences"[Mesh] OR "Maternal-Child Health Centers"[Mesh] OR "Child Welfare"[Mesh] OR "Child Nutritional Physiological Phenomena"[Mesh] OR "Child Nutrition Disorders"[Mesh] OR "Child Day Care Centers"[Mesh] OR "Child Guidance Clinics"[Mesh] OR "Child Mortality"[Mesh] OR "Maternal-Child Nursing"[Mesh] OR "Child Guidance"[Mesh] OR "Child Care"[Mesh] OR "Child Custody"[Mesh] OR "National Institute of Child Health and Human Development (U.S.)"[Mesh] OR "Child Behavior Disorders"[Mesh] OR "Child Reactive Disorders"[Mesh] OR "Child, Orphaned"[Mesh] OR "Child, Preschool"[Mesh] OR "Child, Hospitalized"[Mesh] OR "Child Development Disorders, Pervasive"[Mesh] OR "Child Development"[Mesh] OR "Infant"[Mesh] OR "Infant Mortality"[Mesh] OR "Pediatrics"[Mesh] OR "Pediatric Nursing"[Mesh] OR "Hospitals, Pediatric"[Mesh] OR "Intensive Care Units, Pediatric"[Mesh] OR "Pediatric Dentistry"[Mesh] OR "Pediatric Assistants"[Mesh] OR "Pediatrics"[Mesh] OR "Neonatology"[Mesh] OR "Perinatology"[Mesh] OR "Pediatric Nursing"[Mesh]OR child*[Title/Abstract] OR pediatr* [Title/Abstract] OR paediatr* [Title/Abstract] OR infan* [Title/Abstract]

DISEASES

1. Malnutrition

"Malnutrition"[Mesh] OR "Protein-Energy Malnutrition"[Mesh] OR "Child Nutrition Disorders"[Mesh] OR "Infant Nutrition Disorders"[Mesh] OR "Nutritional Status"[Mesh] OR "Child Nutrition Sciences"[Mesh] OR "Nutrition Assessment"[Mesh] OR "Nutrition Therapy"[Mesh] OR malnutrit* [Title/Abstract] OR Undernutrit*[Title/Abstract] OR Under-nutrit*[Title/Abstract]

2. HIV/AIDS

("HIV Seropositivity"[Mesh] OR "HIV Enteropathy"[Mesh] OR "HIV Antigens"[Mesh] OR "Anti-HIV Agents"[Mesh] OR "HIV Antibodies"[Mesh] OR "HIV Seronegativity"[Mesh] OR "HIV Infections"[Mesh] OR "HIV Seroprevalence"[Mesh] OR "HIV-2"[Mesh] OR "HIV-1"[Mesh]) OR ("Acquired Immunodeficiency Syndrome"[Mesh] OR "AIDS Serodiagnosis"[Mesh] OR "AIDS Vaccines"[Mesh] OR "SAIDS Vaccines"[Mesh]) OR HIV [Title/Abstract] OR AIDS [Title/Abstract] OR "Acquired Immunodeficiency Syndrome" [Title/Abstract] OR HIV/AIDS [Title/Abstract]

3. Diarrhea

"Diarrhea"[Mesh] OR "Diarrhea, Infantile"[Mesh] OR "Rotavirus Infections"[Mesh] OR "Foodborne Diseases"[Mesh] OR "Antidiarrheals"[Mesh] OR "Enteropathogenic Escherichia coli"[Mesh] OR "Shiga-Toxigenic Escherichia coli"[Mesh] OR "Enterotoxigenic Escherichia coli"[Mesh] OR Diarrh*[Title/Abstract]

4. Pneumomia

"Pneumonia"[Mesh] OR "Pneumonia, Bacterial"[Mesh] OR "Pneumonia, Viral"[Mesh] OR pneumonia* [Title/Abstract]

5. Malaria

"Malaria"[Mesh] OR "Malaria, Vivax"[Mesh] OR "Malaria, Cerebral"[Mesh] OR "Malaria, Falciparum"[Mesh] OR "Malaria Vaccines"[Mesh] OR "Acute malaria" [Supplementary Concept] OR "Blackwater Fever"[Mesh] OR malaria* [Title/Abstract]

6. Measles

"Measles"[Mesh] OR "Measles-Mumps-Rubella Vaccine"[Mesh] OR "Measles Vaccine"[Mesh] OR "Measles virus"[Mesh] OR "Subacute Sclerosing Panencephalitis"[Mesh] OR measles [Title/Abstract]

7. Neonatal disorders

((((("Infant, Newborn, Diseases"[Mesh]) OR "Asphyxia Neonatorum"[Mesh]) OR ("Sepsis"[Mesh] OR "Systemic Inflammatory Response Syndrome"[Mesh])) OR "Premature Birth"[Mesh]) OR ("Fetal Membranes, Premature Rupture"[Mesh] OR "Obstetric Labor, Premature"[Mesh])) OR ("Tetanus"[Mesh] OR "Diphtheria-Tetanus-Pertussis Vaccine"[Mesh] OR "Diphtheria-Tetanus-acellular Pertussis Vaccines"[Mesh] OR "Tetanus Toxoid"[Mesh]) OR Neonatal dis* [Title/Abstract] OR Birth asphyxia [Title/Abstract] OR "neonatal asphyxia" [Title/Abstract] OR sepsis [Title/Abstract] OR "preterm birth" [Title/Abstract] OR "preterm delivery" [Title/Abstract] OR "preterm labor" [Title/Abstract] OR "preterm labour" [Title/Abstract] OR tetan* [Title/Abstract]

8. INJURY/ACCIDENTS: NOT INCLUDED

("Accidents"[Mesh]) OR "Wounds and Injuries"[Mesh] OR Injur* [Title/Abstract] OR accident* [Title/Abstract]

#46	Search #1 AND #9 AND ("2011/01/01"[PDAT] : "2011/12/31"[PDAT])	04:16:10	5751
#45	Search #1 AND #9 AND ("2010/01/01"[PDAT] : "2010/12/31"[PDAT])	04:13:17	11962
#44	Search #1 AND #9 AND ("2009/01/01"[PDAT] : "2009/12/31"[PDAT])	04:09:44	11442
#43	Search #1 AND #9 AND ("2008/01/01"[PDAT] : "2008/12/31"[PDAT])	04:06:17	10795
#42	Search #1 AND #9 AND ("2007/01/01"[PDAT] : "2007/12/31"[PDAT])	04:02:44	10354
#41	Search #1 AND #9 AND ("2006/01/01"[PDAT] : "2006/12/31"[PDAT])	03:59:14	9677
#40	Search #1 AND #9 AND ("2005/01/01"[PDAT] : "2005/12/31"[PDAT])	03:56:04	8981
#39	Search #1 AND #9 AND ("2004/01/01"[PDAT] : "2004/12/31"[PDAT])	03:52:20	8405
#38	Search #1 AND #9 AND ("2003/01/01"[PDAT] : "2003/12/31"[PDAT])	03:48:58	8023
#37	Search #1 AND #9 AND ("2002/01/01"[PDAT] : "2002/12/31"[PDAT])	03:45:35	7573
#36	Search #1 AND #9 AND ("2001/01/01"[PDAT] : "2001/12/31"[PDAT])	03:43:17	7331
#35	Search #1 AND #9 AND ("2000/01/01"[PDAT] : "2000/12/31"[PDAT])	03:40:49	7422
#34	Search #1 AND #9 AND ("1999/01/01"[PDAT] : "1999/12/31"[PDAT])	03:37:53	6955
#33	Search #1 AND #9 AND ("1998/01/01"[PDAT] : "1998/12/31"[PDAT])	03:35:33	7065
#32	Search #1 AND #9 AND ("1997/01/01"[PDAT] : "1997/12/31"[PDAT])	03:33:02	6794
#31	Search #1 AND #9 AND ("1996/01/01"[PDAT] : "1996/12/31"[PDAT])	03:26:22	6430
#30	Search #1 AND #9 AND ("1995/01/01"[PDAT] : "1995/12/31"[PDAT])	03:23:56	6912
#29	Search #1 AND #9 AND ("1994/01/01"[PDAT] : "1994/12/31"[PDAT])	03:18:36	6591
#28	Search #1 AND #9 AND ("1993/01/01"[PDAT] : "1993/12/31"[PDAT])	03:16:40	6793
#27	Search #1 AND #9 AND ("1992/01/01"[PDAT] : "1992/12/31"[PDAT])	03:14:06	6437
#26	Search #1 AND #9 AND ("1991/01/01"[PDAT] : "1991/12/31"[PDAT])	03:11:45	6020
#25	Search #1 AND #9 AND ("1990/01/01"[PDAT] : "1990/12/31"[PDAT])	03:09:38	6067
#24	Search #1 AND #9 AND ("1982/01/01"[PDAT] : "1982/12/31"[PDAT])	03:08:05	4870
#22	Search #1 AND #9 AND ("1989/01/01"[PDAT] : "1989/12/31"[PDAT])	03:03:46	5706
#21	Search #1 AND #9 AND ("1988/01/01"[PDAT] : "1988/12/31"[PDAT])	03:00:07	5402
#20	Search #1 AND #9 AND ("1987/01/01"[PDAT] : "1987/12/31"[PDAT])	02:43:23	4781
#19	Search #1 AND #9 AND ("1986/01/01"[PDAT] : "1986/12/31"[PDAT])	02:42:08	4809
#18	Search #1 AND #9 AND ("1985/01/01"[PDAT] : "1985/12/31"[PDAT])	02:40:55	4896
#17	Search #1 AND #9 Limits: Publication Date from 1984/01/01 to 1984/12/31	02:38:12	4519
#16	Search #1 AND #9 Limits: Publication Date from 1983/01/01 to 1983/12/31	02:35:40	4796
#15	Search #1 AND #9 Limits: Publication Date from 1981/01/01 to 1981/12/31	02:32:53	4482
#14	Search #1 AND #9 Limits: Publication Date from 1980/01/01 to 1980/12/31	02:31:26	4468

#10 Search #1 AND #9 02:14:48 285473

#9 Search #2 OR #3 OR #4 OR #5 OR #6 OR #7 OR #8 02:14:10 1035015

#8 Search (((((“Infant, Newborn, Diseases”[Mesh]) OR “Asphyxia Neonatorum”[Mesh]) OR (“Sepsis”[Mesh] OR “Systemic Inflammatory Response Syndrome”[Mesh])) OR “Premature Birth”[Mesh]) OR (“Fetal Membranes, Premature Rupture”[Mesh] OR “Obstetric Labor, Premature”[Mesh])) OR (“Tetanus”[Mesh] OR “Diphtheria-Tetanus-Pertussis Vaccine”[Mesh] OR “Diphtheria-Tetanus-acellular Pertussis Vaccines”[Mesh] OR “Tetanus Toxoid”[Mesh]) OR Neonatal dis* [Title/Abstract] OR Birth asphyxia [Title/Abstract] OR “neonatal asphyxia” [Title/Abstract] OR sepsis [Title/Abstract] OR “preterm birth” [Title/Abstract] OR “preterm delivery” [Title/Abstract] OR “preterm labor” [Title/Abstract] OR “preterm labour” [Title/Abstract] OR tetan* [Title/Abstract]
02:12:06 289762

#7 Search “Measles”[Mesh] OR “Measles-Mumps-Rubella Vaccine”[Mesh] OR “Measles Vaccine”[Mesh] OR “Measles virus”[Mesh] OR “Subacute Sclerosing Panencephalitis”[Mesh] OR measles [Title/Abstract]
02:11:39 22052

#6 Search “Malaria”[Mesh] OR “Malaria, Vivax”[Mesh] OR “Malaria, Cerebral”[Mesh] OR “Malaria, Falciparum”[Mesh] OR “Malaria Vaccines”[Mesh] OR “Acute malaria” [Supplementary Concept] OR “Blackwater Fever”[Mesh] OR malaria* [Title/Abstract]
02:11:01 59473

#5 Search “Pneumonia”[Mesh] OR “Pneumonia, Bacterial”[Mesh] OR “Pneumonia, Viral”[Mesh] OR pneumonia* [Title/Abstract]
02:10:46 129230

#4 Search “Diarrhea”[Mesh] OR “Diarrhea, Infantile”[Mesh] OR “Rotavirus Infections”[Mesh] OR “Foodborne Diseases”[Mesh] OR “Antidiarrheals”[Mesh] OR “Enteropathogenic Escherichia coli”[Mesh] OR “Shiga-Toxigenic Escherichia coli”[Mesh] OR “Enterotoxigenic Escherichia coli”[Mesh] OR Diarrh*[Title/Abstract]
02:10:15 100664

#3 Search (“HIV Seropositivity”[Mesh] OR “HIV Enteropathy”[Mesh] OR “HIV Antigens”[Mesh] OR “Anti-HIV Agents”[Mesh] OR “HIV Antibodies”[Mesh] OR “HIV Seronegativity”[Mesh] OR “HIV Infections”[Mesh] OR “HIV Seroprevalence”[Mesh] OR “HIV-2”[Mesh] OR “HIV-1”[Mesh]) OR (“Acquired Immunodeficiency Syndrome”[Mesh] OR “AIDS Serodiagnosis”[Mesh] OR “AIDS Vaccines”[Mesh]

OR “SAIDS Vaccines”[Mesh]) OR HIV [Title/Abstract] OR AIDS [Title/Abstract] OR “Acquired Immunodeficiency Syndrome” [Title/Abstract] OR HIV/AIDS [Title/Abstract]
02:09:54 292013

#2 Search “Malnutrition”[Mesh] OR “Protein-Energy Malnutrition”[Mesh] OR “Child Nutrition Disorders”[Mesh] OR “Infant Nutrition Disorders”[Mesh] OR “Nutritional Status”[Mesh] OR “Child Nutrition Sciences”[Mesh] OR “Nutrition Assessment”[Mesh] OR “Nutrition Therapy”[Mesh] OR malnutrit* [Title/Abstract] OR Undernutrit*[Title/Abstract] OR Under-nutrit*[Title/Abstract]
02:09:19 199987

#1 Search “Child”[Mesh] OR “Child Health Services”[Mesh] OR “Child Nutrition Sciences”[Mesh] OR “Maternal-Child Health Centers”[Mesh] OR “Child Welfare”[Mesh] OR “Child Nutritional Physiological Phenomena”[Mesh] OR “Child Nutrition Disorders”[Mesh] OR “Child Day Care Centers”[Mesh] OR “Child Guidance Clinics”[Mesh] OR “Child Mortality”[Mesh] OR “Maternal-Child Nursing”[Mesh] OR “Child Guidance”[Mesh] OR “Child Care”[Mesh] OR “Child Custody”[Mesh] OR “National Institute of Child Health and Human Development (U.S.)”[Mesh] OR “Child Behavior Disorders”[Mesh] OR “Child Reactive Disorders”[Mesh] OR “Child, Orphaned”[Mesh] OR “Child, Preschool”[Mesh] OR “Child, Hospitalized”[Mesh] OR “Child Development Disorders, Pervasive”[Mesh] OR “Child Development”[Mesh] OR “Infant”[Mesh] OR “Infant Mortality”[Mesh] OR “Pediatrics”[Mesh] OR “Pediatric Nursing”[Mesh] OR “Hospitals, Pediatric”[Mesh] OR “Intensive Care Units, Pediatric”[Mesh] OR “Pediatric Dentistry”[Mesh] OR “Pediatric Assistants”[Mesh] OR “Pediatrics”[Mesh] OR “Neonatology”[Mesh] OR “Perinatology”[Mesh] OR “Pediatric Nursing”[Mesh] OR child*[Title/Abstract] OR pediatr* [Title/Abstract] OR paediatr* [Title/Abstract] OR infan* [Title/Abstract]
02:08:31 2122246
